# Adjunctive Nutritional Supplementation in Drug-Resistant Epilepsy: A Prospective Observational Study Evaluating Quality of Life and Seizure Outcomes

**DOI:** 10.7759/cureus.105895

**Published:** 2026-03-26

**Authors:** Anoop K Singh, Gayatri Kumari

**Affiliations:** 1 Department of Neurosurgery, Lifeline Hospital and Research Centre, Azamgarh, IND; 2 Department of Anesthesiology and Critical Care, Lifeline Hospital and Research Centre, Azamgarh, IND

**Keywords:** dietary supplements, epilepsy, quality of life, refractory, seizures

## Abstract

Introduction: Drug-resistant or refractory epilepsy remains a significant clinical challenge, with persistent seizures and impaired quality of life, despite optimized antiepileptic therapy. Increasing attention has been directed toward adjunctive nutritional strategies that may support neuronal stability and overall well-being. The aim of the present study was to evaluate the real-world impact of Epregres Unique 12 as an adjunct to standard antiepileptic drug therapy on the quality of life and seizure frequency in patients with drug-resistant epilepsy.

Materials and methods: This prospective observational study was conducted at a tertiary care center between January 2023 and December 2024. Twenty-six patients with drug-resistant epilepsy were followed for 12 weeks after the initiation of adjunctive nutritional supplementation. Antiepileptic drug regimens were continued without protocol-driven modifications. The quality of life was assessed using the Quality of Life in Epilepsy-31 (QOLIE-31) questionnaire at baseline and after 12 weeks. Seizure frequency was documented by using patient-maintained seizure diaries. Data were analyzed using paired statistical tests with effect size estimation.

Results: Twenty-six patients (19 males, seven females; mean age 37.2 ± 7.4 years) were included. Emotional well-being showed a statistically significant improvement at 12 weeks (p = 0.009) with a moderate effect size. The total QOLIE-31 score demonstrated a clinically meaningful improvement, approaching statistical significance (p = 0.061). Seizure frequency decreased in 20 (76.9%) patients, including complete seizure cessation in 6 (23.1%) patients. The supplement was well-tolerated, with only one minor adverse event reported.

Conclusion: Adjunctive nutritional supplementation is associated with improvements in emotional well-being and favorable trends in quality of life and seizure frequency in patients with drug-resistant epilepsy. While these findings are encouraging, controlled studies with larger sample sizes are required to confirm these observations.

## Introduction

Epilepsy is a chronic neurological disorder characterized by recurrent unprovoked seizures, and remains one of the most common disorders of the central nervous system worldwide [1]. Despite the availability of multiple antiepileptic drugs (AEDs), nearly 11.3% of patients continue to experience seizures and are classified as having drug-resistant epilepsy (DRE) or refractory epilepsy, as per the data available from the East Indian population [2]. These patients often face persistent seizure burden, cognitive decline, psychosocial stress, and a reduced quality of life (QoL). The long-term use of multiple AEDs further adds to adverse effects, drug interactions, and economic strain, underscoring the need for safe and supportive adjunctive approaches [3].

In recent years, interest in nutritional and metabolic interventions that may complement conventional pharmacotherapy has grown. Nutrients, such as omega-3 fatty acids, coenzyme Q10, taurine, B-complex vitamins, vitamin D, and trace minerals, have been studied for their neuroprotective, antioxidant, and membrane-stabilizing properties [4]. These agents are believed to influence mitochondrial function, oxidative stress pathways, and inhibitory neurotransmission, mechanisms that are increasingly being recognized in the pathophysiology of refractory seizures [5]. However, clinical evidence regarding structured multi-nutrient formulations in DRE remains limited and controversial, particularly in real-world clinical settings [6].

Unique 12 is a sugar-free liquid nutritional supplement formulated using a combination of neuro-supportive micronutrients. It contains a blend of omega-3 fatty acids, CoQ10, taurine, vitamin B12, folic acid, vitamin D3, and minerals, such as zinc, magnesium, and selenium. Given the paucity of systematic observational data on its use in patients with DRE, evaluation under routine clinical practice conditions was considered relevant. Therefore, this prospective observational study was conducted to assess the impact of Epregres Unique 12 as an adjunct to standard therapy in patients with DRE. The primary objective was to evaluate changes in quality of life (QoL) over 12 weeks. The secondary objectives included the assessment of seizure frequency trends, emotional well-being, cognitive domains, and tolerability.

## Materials and methods

This prospective observational study was conducted at Lifeline Hospital and Research Center, Azamgarh, India, between January 2023 and December 2024. Each enrolled participant was followed up for 12 weeks from the initiation of Epregres Unique 12 supplementation. The observational design indicated that there was no randomization, placebo control, or protocol-driven alteration of the standard therapy. Epregres Unique 12 was prescribed as part of routine adjunctive clinical care, and investigators documented outcomes without interfering with the ongoing AED regimens. All treatment decisions, including AED selection and dose adjustments, were at the discretion of the treating neurologist, thereby reflecting real-world clinical practice. Ethical approval was obtained from the Institutional Ethics Committee (Ref. No. ECR/LH/2023/014, dated 10/01/23), and written informed consent was obtained from all adult participants and from parents or legal guardians in the case of minors prior to enrollment.

Patients attending the outpatient department who met the definition of DRE as failure of seizure control despite adequate trials of at least two appropriately chosen and tolerated AEDs were screened consecutively [3]. Eligible participants were aged 15-60 years and on stable AED therapy for at least four weeks prior to enrollment. Patients with metabolic disorders affecting nutrient absorption, pregnancy or lactation, known hypersensitivity to supplement components, or an inability to comply with follow-up were excluded.

Sample size was estimated using G*Power (Heinrich Heine University, Düsseldorf, Düsseldorf, Germany). Based on a clinically meaningful effect size of 0.6 for paired comparison of QOLIE-31 scores, a minimum of 22 participants were required to achieve 80% power at a 5% alpha error [7]. Accounting for a 20% attrition rate, 26 patients were enrolled in the study.

The participants received Epregres Unique 12, a sugar-free liquid supplement, as an adjunct to their existing AED regimen for 12 weeks. The supplement was administered orally once daily with meals following a standardized weight-based dosage of 0.25 mL/kg/day (10 mL daily for patients weighing ≤40 kg and 20 mL daily for those weighing >40 kg). The AED regimens were not modified as part of the study protocol unless clinically indicated. Compliance was assessed during the monthly follow-up visits.

Baseline demographic and clinical details, including age, sex, seizure type, duration of epilepsy, neuroimaging findings, and number of AEDs, were recorded. Patients and caregivers maintained daily seizure diaries documenting seizure frequency, type, and duration. Monthly visits were conducted to review the diary entries and document adverse events.

Quality of life was assessed using the Quality of Life in Epilepsy-31 (QOLIE-31) questionnaire (RAND Health Care, Santa Monica, California, USA) [8]. The instrument is publicly available and free for non-commercial research use on the official website, along with its scoring manual. The questionnaire was administered at baseline and after 12 weeks.

Data entry was performed using Microsoft Excel 2013 (Microsoft Corporation, Redmond, WA). Statistical analyses were performed using the statistical software (IBM SPSS Statistics, version 21.0; IBM Corp., Armonk, New York, USA). Descriptive statistics were used to summarize demographic and clinical characteristics. Pre- and post-treatment QOLIE-31 scores were compared using paired Student’s t-tests after the assessment of normality. Statistical significance was set at P < 0.05. The effect sizes (Cohen's d) were calculated to determine the magnitude of the change. Seizure frequency trends were analyzed descriptively owing to the heterogeneity in the baseline seizure burden.

## Results

A total of 26 patients with DRE were included in this study. The baseline demographic and clinical characteristics of the patients are presented in Table 1. The mean age of the participants was 37.2 ± 7.4 years (range 15-55 years). The cohort consisted predominantly of males, with females accounting for seven (26.9%). Generalized tonic-clonic seizures were the most common seizure type, followed by complex partial seizures with or without secondary generalization, and juvenile myoclonic epilepsy. Neuroimaging abnormalities were identified in 15 (57.7%) patients, and 11 (42.3%) had normal imaging findings. Participants had a mean of 2.0 ± 0.6 AEDs, and the mean duration of epilepsy was 8.4 ± 5.3 years.

**Table 1:** Baseline demographic and clinical characteristics of patients with drug-resistant epilepsy (N = 26). Values are presented as mean ± standard deviation (SD) or number (percentage). *Abnormal neuroimaging findings included periventricular leukomalacia, hippocampal sclerosis, encephalomalacia, calcified granulomas, and gliosis. AED: antiepileptic drug.

Characteristic	Value
Age (years)	
Mean ± SD	37.2 ± 7.4
Range	15–55
Sex, n (%)	
Male	19 (73.1)
Female	7 (26.9)
Seizure type, n (%)	
Generalized tonic-clonic	23 (88.5)
Juvenile myoclonic epilepsy	1 (3.8)
Complex partial ± secondary generalization	2 (7.7)
Neuroimaging findings, n (%)	
Normal	11 (42.3)
Abnormal*	15 (57.7)
Number of antiepileptic drugs	
Mean ± SD	2.0 ± 0.6
Range	1–3
Duration of epilepsy (years)	
Mean ± SD	8.4 ± 5.3
Range	0.8–19

During the 12-week observational follow-up period, seizure diary analysis demonstrated a reduction in seizure frequency in 20 (76.9%) patients. Complete seizure cessation during the study period was documented in 6 (23.1%) patients. Seizure frequency remained unchanged in 4 (15.4%) patients, while 2 (7.7%) patients had incomplete documentation, limiting detailed trend assessment. Due to the variability in baseline seizure burden and heterogeneity of seizure patterns, seizure frequency outcomes have been reported descriptively. The supplement was well-tolerated during the 12-week follow-up period. Only 1 (3.8%) patient reported mild gastrointestinal discomfort, which resolved without discontinuation. Serious adverse events were not observed.

Pre- and post-treatment comparisons of the QOLIE-31 domain scores are summarized in Table 2. Improvement in emotional well-being was statistically significant (p = 0.009), with mean scores increasing from 7.52 ± 1.53 to 8.71 ± 1.61. Domain-level improvement was observed in 18 (69.2%) patients. The total QOLIE-31 score increased from 48.29 ± 16.25 at baseline to 55.44 ± 9.31 at 12 weeks, with improvement observed in 19 (73.1%) patients. Although the paired-comparison approach approached statistical significance (p = 0.061), the effect size indicated a clinically meaningful change. The other domains showed positive trends. Improvement in seizure worry was observed in 15 (57.7%) patients; overall QoL in 17 (65.4%) patients; cognitive function in 16 (61.5%) patients; social functioning in 17 (65.4%) patients; and medication effects in 14 (53.8%) patients. Energy/fatigue showed improvement in 9 (34.6%) patients, but did not reach statistical significance. Overall, adjunctive supplementation with Epregres Unique 12 was associated with favorable trends in seizure reduction and statistically significant improvements in emotional well-being, along with clinically meaningful enhancements in overall QoL.

**Table 2:** Comparison of pre- and post-treatment QOLIE-31 scores after 12 weeks of adjunctive supplementation (N = 26). Values are presented as mean ± standard deviation (SD). Paired Student's t-test was used for comparison. *Statistically significant at p < 0.05. Cohen's d represents effect size (0.2 = small, 0.5 = moderate, 0.8 = large effect).

Domain	Pre-treatment (mean ± SD)	Post-treatment (mean ± SD)	Mean difference	t-value	p-value	Cohen's d
Seizure worry	3.60 ± 2.57	4.26 ± 1.81	–0.67	–1.069	0.290	0.21
Overall quality of life	6.54 ± 4.98	8.13 ± 3.11	–1.59	–1.362	0.179	0.27
Emotional well‑being	7.52 ± 1.53	8.71 ± 1.61	–1.19	–2.707	0.009*	0.54
Energy/fatigue	6.14 ± 1.15	6.29 ± 1.23	–0.15	–0.448	0.656	0.09
Cognitive function	13.32 ± 6.35	15.31 ± 4.13	–1.99	–1.323	0.192	0.26
Medication effects	1.46 ± 0.52	1.68 ± 0.58	–0.22	–1.434	0.158	0.29
Social function	9.71 ± 3.07	11.05 ± 3.04	–1.34	–1.561	0.125	0.31
Total QOLIE‑31 score	48.29 ± 16.25	55.44 ± 9.31	–7.15	–1.917	0.061	0.38

## Discussion

Despite the availability of multiple antiepileptic drugs, DRE remains a major therapeutic challenge. Even with rational polytherapy, a considerable proportion of patients continue to experience persistent seizures with emotional, cognitive, and psychosocial consequences. The present prospective observational study explored the real-world use of Epregres Unique 12 as an adjunct to standard therapy and provided clinically relevant insights into its potential supportive role.

The most notable finding in this cohort was the significant improvement in emotional well-being. Mood disturbances are highly prevalent in patients with epilepsy and are often underrecognized. Depression and anxiety not only reduce quality of life but may also lower the seizure threshold and impair treatment adherence [3]. Nutritional components, such as omega-3 fatty acids, B-complex vitamins (particularly pyridoxine), vitamin D, and magnesium, have been individually associated with improved mood regulation and neurochemical balance in previous studies [5,6,9]. Recent evidence supports the psychotropic role of omega-3 polyunsaturated fatty acids. Serefko et al. [10] highlighted that omega-3 polyunsaturated fatty acids exert antidepressant effects by modulating inflammatory pathways, regulating monoaminergic neurotransmission, and improving neuronal membrane fluidity. The authors emphasized that eicosapentaenoic acid-dominant formulations may reduce depressive symptom severity, particularly in individuals with elevated inflammatory markers, such as interleukin-6, tumor necrosis factor-alpha, and C-reactive protein. These biological mechanisms may partly explain the improvements observed in the emotional well-being domain in the present study [10].

Vitamin D deficiency has also been linked to depressive symptoms and increased seizure frequency in epilepsy populations [11]. Therefore, the observed improvement in emotional well-being in this study may reflect the cumulative neurobiological effects of these micronutrients on neurotransmitter modulation and inflammatory pathways. Although the overall QOLIE-31 scores did not reach conventional statistical significance, the magnitude of improvement and effect size suggested clinically meaningful changes. This is important because QoL measures often capture subtle yet functionally relevant improvements that may not always translate into statistically robust findings in small cohorts. Previous observational and interventional studies evaluating omega-3 supplementation in epilepsy have reported reductions in seizure frequency and improvements in cognitive alertness, although the results have been inconsistent [6,12]. Similarly, studies exploring coenzyme Q10 supplementation have highlighted its role in mitochondrial stabilization and reduction of oxidative stress, mechanisms increasingly implicated in refractory epilepsy [13,14]. The direction of the change observed in the present study aligns with this emerging body of literature, suggesting that mitochondrial and antioxidant support may complement pharmacological seizure control.

The reduction in seizure frequency observed descriptively in the majority of patients is encouraging, although it must be interpreted cautiously. Earlier trials of isolated omega-3 fatty acid supplementation have shown mixed outcomes, with some demonstrating modest seizure reduction and others reporting neutral results [6,12]. One possible explanation is that multi-nutrient formulations may exert synergistic effects that cannot be achieved by single-agent supplementation. For example, taurine has demonstrated anticonvulsant properties in experimental models by modulating calcium channels and enhancing inhibitory neurotransmission [15]. When combined with antioxidants and micronutrients that support neuronal metabolism, a broader stabilizing effect on neuronal excitability may occur. Although causality cannot be established in an observational framework, the consistency of seizure reduction trends across different seizure types in this cohort suggests that adjunctive metabolic support warrants further structured investigation.

The favorable tolerability profile observed in this study is clinically relevant. Polypharmacy is common in DRE, and additional pharmacological agents often increase the burden of adverse effects [16]. The absence of serious adverse events and high satisfaction rates indicated that adjunctive nutritional supplementation may be acceptable to patients and caregivers. In chronic neurological conditions, adherence and perceived benefit play a crucial role in long-term management outcomes [17,18]. Ran et al. [19] reported significant associations between dietary nutrient intake and epilepsy prevalence in a cross-sectional analysis of the National Health and Nutrition Examination Survey (2013-2014). The study suggested that inadequate intake of certain micronutrients may be linked to a higher epilepsy risk, supporting the potential relevance of nutritional optimization in epilepsy management.

From a clinical perspective, these findings suggest that adjunctive micronutrient supplementation may serve as a supportive strategy in carefully selected patients with DRE, particularly in those with comorbid mood disturbances or suspected micronutrient deficiencies. It should not replace established antiepileptic therapies, but may complement them by targeting metabolic and inflammatory pathways not directly addressed by conventional AEDs. Routine screening for vitamin deficiencies and consideration of metabolic optimization may therefore represent a rational addition to comprehensive epilepsy care.

However, this study has several limitations that must be acknowledged. This observational design precludes causal inference and is susceptible to selection bias and confounding factors. The absence of a control group limited the ability to differentiate treatment effects from natural fluctuations in seizure patterns or placebo responses. The relatively small sample size reduced the statistical power for secondary outcomes, and seizure frequency was analyzed descriptively owing to heterogeneity in the baseline documentation. Additionally, follow-up was limited to 12 weeks, preventing the assessment of the long-term sustainability of benefits.

Future research should include adequately powered randomized controlled trials with placebo comparisons to validate these findings. Stratification by seizure type, baseline micronutrient status, and epilepsy duration would help to identify subgroups that are most likely to benefit. Longer follow-up periods are necessary to determine the durability of the effects and safety over time. The incorporation of objective biomarkers such as inflammatory markers, oxidative stress indices, or mitochondrial function assays could further clarify the mechanistic pathways.

## Conclusions

In this prospective observational study, adjunctive use of Epregres Unique 12 in patients with DRE was associated with improvements in emotional well-being and favorable trends in overall QoL and seizure frequency over 12 weeks. While a statistically significant change was observed primarily in the emotional domain, the other domains demonstrated clinically meaningful but modest improvements. The supplement was well-tolerated, with high patient satisfaction and no serious adverse events. Given the observational design and limited sample size, these findings should be interpreted with caution. Larger, well-designed, randomized controlled trials are needed to clarify its efficacy and establish definitive clinical recommendations.
